# Analysis of infection-related adverse events induced by proton pump inhibitors based on the FAERS and JADER databases

**DOI:** 10.1097/MD.0000000000046349

**Published:** 2025-12-05

**Authors:** Shanshan Hu, Hongxiu Yu, Zhi Liu

**Affiliations:** a Department of Pharmacy, Affiliated Hospital of Guizhou Medical University, Guiyang City, Guizhou Province, China; b Department of Pharmacy, Panjiang Coal and Electricity Group Hospital, Liupanshui, Guizhou Province, China; c Department of Dermatology, Affiliated Hospital of Guizhou Medical University, Guiyang, Guizhou Province, China.

**Keywords:** adverse event, data mining, FAERS database, JADER database, proton pump inhibitor, reporting odds ratio

## Abstract

**Background:**

This study aims to analyze infection-related adverse events related to different proton pump inhibitors (PPIs) and explore the characteristics of Infection-related adverse events and the intensity of drug association based on the U.S. Food and Drug Administration adverse event reporting system (FAERS) database and Japan Adverse Drug Event Report Database (JADER), so as to promote the rational application of PPIs.

**Methods:**

The adverse event reports of omeprazole, lansoprazole, pantoprazole, rabeprazole, esomeprazole, dexlansoprazole and vonoprazan in 78 quarters from the first quarter of 2004 to the fourth quarter of 2023 were searched and extracted by FAERS and JADER, and the adverse events were systematically classified by the Medical Dictionary for Regulatory Activities term set. Then, data processing and analysis of the 7 drug-related reports were carried out by reporting odds ratio method.

**Results:**

Inclusion of infection-related adverse event report statistics: in the FAERS database, 2 806 reports of omeprazole, 1 652 reports of lansoprazole, 2 922 reports of pantoprazole, 265 reports of rabeprazole, 6 004 reports of esomeprazole, and 297 reports of dexlansoprazole; and in the JADER database, 79 reports of omeprazole, 137 reports of esomeprazole, 414 reports of lansoprazole, 117 reports of rabeprazole, and 204 reports of vonoprazan; 3 185 adverse drug reactions (ADRs) signals were obtained in the FAERS database with signals involving 27 system organ class, whereas 478 ADRs signals were obtained in the JADER database with ADRs signals involving 25 system organ class, and infectious pneumonias were the most common in both databases.

**Conclusions:**

In the use of PPIs, we should pay attention to the occurrence of related adverse reactions, especially the damage to the infection-related adverse effects, monitor the relevant indicators, and take timely intervention measures to ensure the safety of medication.

## 1. Introduction

The use of proton pump inhibitors (PPIs) is increasing worldwide. Since their introduction in the 1980s, they have become one of the most commonly used classes of drugs worldwide, inhibiting gastric acid secretion by acting on the H^+^/K^+^-ATP enzyme in gastric lining cells.^[1]^ However, PPIs are overused for over-specified indications, excessive doses and long-term treatment.^[2,3]^ The overuse of PPIs is a worldwide problem, and studies have found that more than 20% of cases of PPIs do not have clear indications.^[4]^ In the United States, 25% to 70% of prescriptions for PPIs are not appropriately indicated,^[5,6]^ In a survey of PPIs prescribed in 45 hospitals in China, 32.6% to 56.8% of PPIs were prescribed for unapproved indications,^[2]^ A multicentre observational study 2020 showed that 22% to 63% of PPIs in Italian nursing homes were improperly treated.^[3]^ With the widespread use of PPIs, more and more studies have focused on the safety of PPIs therapy, and the number of common adverse drug reactions (ADRs) and serious ADRs has gradually increased,^[7]^ Including fractures, acute and chronic kidney disease, gastrointestinal infections, vitamin B_12_ and magnesium deficiencies, and respiratory infections,^[8–10]^ One of these studies suggests that the use of PPIs may increase the risk of infections, such as COVID-19 infection,^[11]^ gastrointestinal infection,^[12]^ clostridium difficile infection,^[13]^ respiratory infections^[14]^and so on. However, there is no high or moderate quality evidence to support the association.^[15]^ The link between PPIs use and pneumonia was first reported in 2004 and has been the subject of numerous studies since then.^[16]^ The results of a 2008 meta-analysis showed that the use of PPIs resulted in a low incidence of respiratory infections of about 4%.^[17]^ The results of a retrospective study showed a total of 0.22% pneumonia cases in the esomeprazole group and 0.21% pneumonia cases in the placebo group, which are comparable in incidence, suggesting that esomeprazole use is not associated with the development of pneumonia.^[18]^ Following the outbreak of neocoronitis, the results of the latest meta-analysis showed that the current use of PPIs significantly increased mortality due to neocoronitis, but the development of neocoronitis was not significantly increased.^[11]^ There is insufficient clinical trial data to determine whether there is an association between PPIs and pneumonia, and this article analyses infection-related adverse events (IRAE) caused by PPIs, with a focus on pneumonia, with the help of the ADR Database.

In recent years, large real-world adverse drug event (ADE) databases, such as Food and Drug Administration adverse event reporting system (FAERS) and JADER, have included an increasing number of data.^[19]^ In this study, we used the FAERS and JADER databases. FAERS was constructed by Food and Drug Administration through the collection of ADE cases from around the world and is one of the largest databases available to the public. As of the end of 2019, FAERS has collected more than 10 million cases containing ADE reports submitted by healthcare professionals, manufacturers, consumers, and attorneys. These reports can be quantitatively analyzed using data mining methods to detect signals of drug-related adverse events.^[20]^ JADER was established by the Pharmaceutical and Medical Devices Agency of Japan through the collection of ADE cases since April 2004 in Japan. The 2 databases are reported to have different characteristics. Due to the different characteristics of the databases, their results will be different. Therefore, we analyzed the 2 databases separately and compared the results. By analyzing and comparing the collected information on infection-related adverse events caused by PPIs, we mined their potential signals of adverse events, with a view to optimizing patient treatment protocols and providing a reference for the safe and rational use of medication in the clinic.

## 2. Methods

### 2.1. Source of data

We used 2 large databases of spontaneous adverse drug Search for types of PPIs according to domestic and international guidelines,^[9]^ The FAERS database was locked for omeprazole, lansoprazole, pantoprazole, rabeprazole, esomeprazole, and dexlansoprazole. In Japan, since pantoprazole and dexlansoprazole are not available, the JADER database was locked with the types omeprazole, lansoprazole, rabeprazole, esomeprazole, and vonoprazan. The English generic names of the above drugs were searched and their adverse event reports were extracted for a total of 78 quarters from Q1 2004 to Q2 2023, and then files containing information on ADEs, adverse event reporting dates and results, route of administration, dose administered, cumulative dose of the drug administered, gender and age of the patient, and reporting country were downloaded and imported into Microsoft Excel for the next step.

### 2.2. Data screening and cleansing

Unrecognizable, meaningless drug names, nontarget drugs, and duplicate reports were excluded. Adverse events are encoded according to the Preferred Term in the Medical Dictionary for Regulatory Activities, and are summarized into the system organ class (SOC) according to the structure in the Medical Dictionary for Regulatory Activities23.0 version.

### 2.3. Data analysis

In this study, the reporting odds ratio (ROR) and the comprehensive standard method (MHRA) were used to calculate the ROR value, the proportional reporting ratio (PRR) and the chi-square (χ^2^), respectively, and the potential ADE signals were screened. Based on the 4-fold table of the proportional imbalance method (Table 1), the data such as the number of target ADE reports of the primary suspected drugs and the background number of ADE occurrence were obtained, and the ROR and PRR values were calculated according to the formula (Table 2).^[21]^ Events with 95 % CI lower limit >1 and no <3 cases reported in the ROR method and events with PRR ≥2, χ^2^ ≥ 4and no <3 cases reported in the MHRA method were defined as ADE signals. All statistical analyses were performed using Microsoft Excel 2023 software.

**Table 1:** Fourfold table of disproportional method.

Drugs	Target number of adverse reaction reports	Number of other adverse reaction reports	Total
Target drugs	*a*	*b*	*a*+*b*
Other drugs	*c*	*d*	*c*+*d*
Total	*a*+*c*	*b*+*d*	N = *a*+*b*+*c*+*d*

**Table 2:** Formulas and threshold values of ROR and MHRA.

Methods	Formula	Threshold value
ROR	$ROR=\frac{\left(a/c\right)}{\left(b/d\right)}=\frac{ad}{bc}$ $SE\left(lnROR\right)=\sqrt{\;\;\;\;\;\left(\frac{1}{a}+\frac{1}{b}+\frac{1}{c}+\frac{1}{d}\right)\;\;\;\;\;}$ $95\;\%\;\;CI=e^{ln\left(ROR\right)\pm 1.96\sqrt{\;\;\;\;\;\left(\frac{1}{a}+\frac{1}{b}+\frac{1}{c}+\frac{1}{d}\right)\;\;\;\;\;}}$	*a* ≥3, and 95 % CI (lower limit) >1 indicated that a signal was generated.
MHRA	$PRR\frac{a/\left(a+b\right)}{c/\left(c+d\right)}$ $\chi 2=\frac{\;{\left(ad-bc\right)}^{2}\left(a+b+c+d\right)}{\left(a+b\right)\left(a+c\right)\left(c+d\right)\left(b+d\right)}$	a ≥3, PRR ≥2, χ^2^ ≥4, then prompts to generate 1 signal

MHRA = the comprehensive standard method, PRR = the proportional reporting ratio, ROR = the reporting odds ratio, χ^2^ = the chi-square.

## 3. Results

### 3.1. Adverse event reporting basics

After removing duplicates, a total of 16,623,939 reports were obtained from the FAERS database of adverse events for 78 quarters from Q1 2004 to Q2 2023 for the drug of primary suspicion (ADE). Among them, there were 256,785 ADEs with 67,578 reports for the first suspected drug esomeprazole; 127,209 ADEs with 35,583 reports for the first suspected drug omeprazole; 85,057 ADEs with 25,396 reports for the first suspected drug lansoprazole; 94,555 ADEs with 28,514 reports for the first suspected drug pantoprazole; 28,514 ADEs with 28,514 reports for the first suspected drug pantoprazole; and 16,623,939 reports for the “drug of first suspicion” after deleting duplicate reports. The total number of ADEs in which the drug of primary suspicion was dexlansoprazole was 15,707, with 5593 reports; the total number of ADEs in which the drug of primary suspicion was rabeprazole was 8635, with 3295 reports.

With 78 quarters of adverse event data from the JADER database from Q1 2004 to Q2 2023, we identified 13,072 users of esomeprazole, 9623 users of omeprazole, 38,635 users of lansoprazole, 13,255 users of rabeprazole, and 10,148 users of vonoprazan. A total of 2642 ADEs for esomeprazole were reported in 1593 cases, 1869 ADEs for omeprazole in 1148 cases, 7565 ADEs for lansoprazole in 4776 cases, 2430 ADEs for rabeprazole in 1705 cases, and 3097 ADEs for vonoprazan in 2021 cases.

The number of IRAE reports caused by PPIs was further determined to be 13 946 in the FAERS database and 951 in the JADER database. Among them, esomeprazole caused the most IRAE in the FAERS database, accounting for 42.05 %, while lansoprazole caused the most IRAE in the JADER database, accounting for 43.63 %. In the age-reported cases, both databases suggested that the proportion of patients over 45 years old using PPIs to cause IRAE was greater than that of patients of other age groups. The number of female cases reported in FAERS database was more than that of male cases, and the number of male cases in JADER database was more than that of female cases, with the ratio of male to female being 2.05 and 2.20, respectively. In the FAERS database, consumers reported the most cases (39.84%), followed by doctors (17.18%) and pharmacists (12.37%), while in the JADER database, doctors reported the most cases (65.51%), followed by other health personnel (20.93%) and pharmacists (7.78%). In the FAERS database, the United States reported the most cases (49.4%), followed by Canada (10.14%) and the United Kingdom (7.50%), while all the data in the JADER database were from Japan. The basic information such as the classification of the number of reports, the reporting country, the patient’s gender and age are shown in Table 3.

**Table 3:** Basic status of infection-related adverse event reports in the database.

Characteristics	Subgroups	IRAE			
		FAERS		JADER	
		Cases/N	Proportion/%	Cases/N	Proportion/%
Cases	All	13 946	100.00	951	100.00
	Omeprazole	2 806	20.12	79	8.31
	Esomeprazole	6 004	42.05	137	14.41
	Lansoprazole	1 652	11.85	414	43.53
	Dexlansoprazole	297	2.13	–	–
	Pantoprazole	2 922	20.95	–	–
	Rabeprazole	265	1.90	117	12.30
	Vonoprazan	–	–	204	21.45
Sex	Male	4 283	30.70	515	54.15
	Female	8 792	63.04	234	24.61
	Unknown	874	6.26	202	21.24
Ages	<18 yr	252	1.81	18	1.89
	18–45 yr	1403	10.06	70	7.36
	45–65 yr	4583	32.86	345	36.28
	65–75 yr	2364	16.95	257	27.02
	≥75 yr	2381	17.07	237	24.93
	Unknown	2 963	21.25	24	2.52
Reporters	Lawyers	277	1.99	–	–
	Physician	2 396	17.18	623	65.51
	Pharmacist	1 725	12.37	74	7.78
	Consumers	5 557	39.84	25	2.63
	Other health professional	1 440	10.33	199	20.93
	Unknown	2 551	18.29	30	3.15
Countries (top5)	United States	6 890	49.40	Japan:951	
	Canada	1 414	10.14		
	Britain	1 053	7.55		
	France	909	6.52		
	Germany	576	4.13		
Years	2020	1 141	8.18	82	8.63
	2021	1 092	7.83	88	9.25
	2022	952	6.83	64	6.73
	2023	771	5.53	–	–
	2004–2019	9 990	71.63	717	75.39

FAERS = the U.S. Food and Drug Administration adverse event reporting system, IRAE = infection-related adverse effects, JADER** **=** **the Japan adverse drug event report database, N** **=** **number.

### 3.2. System organs involved in ADE reports and ADRs signals

**Figure 1.**
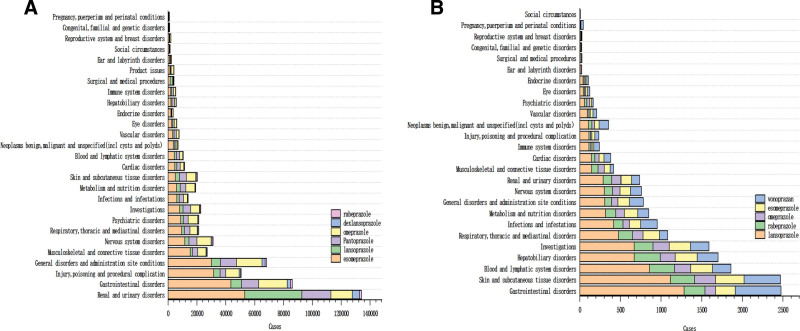
(A) The SOC of ADRs signals of PPIs drugs in the FAERS database, (B) The SOC of ADRs signals of acid-suppressing drugs in the JADER database. ADRs = adverse drug reactions, FAERS = FDA adverse event reporting system, PPIs = proton pump inhibitors, SOC = the system organ class.

**Figure 2.**
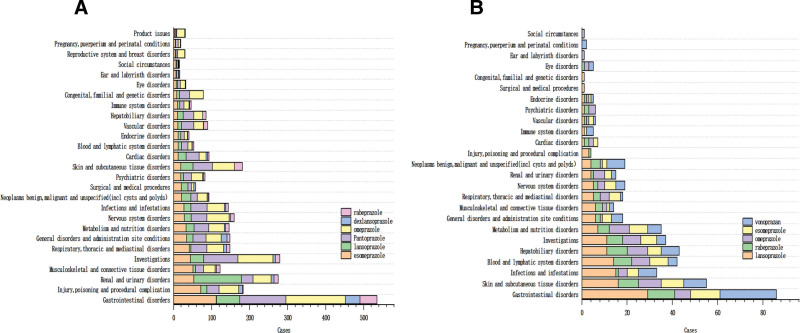
(A) The SOC of ADRs signals of PPIs drugs in the FAERS database, (B) The SOC of ADRs signals of PPIs drugs in the JADER database. ADRs = adverse drug reactions, FAERS = FDA adverse event reporting system, JADER = Japan adverse drug event report database, PPIs = proton pump inhibitors, SOC = the system organ class.

The ADE report of PPIs drugs involved 27 SOCs, of which the top 3 in the FAERS database were kidney and urinary system diseases, gastrointestinal system diseases and systemic diseases and various reactions at the site of administration, as shown in Figure 1A. In the JADER database, the top 3 were gastrointestinal system, skin and subcutaneous tissue, and blood and lymphatic system diseases, as shown in Figure 1B. After mining by ROR and PRR methods, 3185 ADRs signals were obtained in the FAERS database, including 923 omeprazole, 776 pantoprazole, 678 esomeprazole, 494 lansoprazole, 194 rabeprazole, and 120 dexlansoprazole; the ADRs signals were related to 27 SOCs, and the most common one was the gastrointestinal disorders, with all kinds of examinations in the second place, followed by renal and urological diseases, skin and subcutaneous tissue diseases and various neurological diseases, the results are shown in Figure 2A. Whereas 478 ADRs signals were obtained from the JADER database, of which 143 were for lansoprazole, 96 were for vonoprazan, 83 were for esomeprazole, 82 were for omeprazole, and 74 were for rabeprazole. the ADRs signals were related to 25 SOCs, the most common being gastrointestinal system disorders, skin and subcutaneous tissue disorders were in the second place, followed by hepatobiliary system disorders, blood and lymphatic system disorders with various types of tests, the results are shown in Figure 2B.

### 3.3. Reporting and signaling classification of infection-related adverse events

According to the site of infection and the pathogenic bacteria, the classification was carried out. Among them, respiratory tract infection was the most common in the FAERS database, and bacterial infection was the most common, accounting for 39.43 % and 34.26 %, respectively. Respiratory tract and bacterial infections were also the most common in the JADER database, accounting for 32.80 % and 44.79 %, respectively. According to the number of IRAE reports, the infection ranked first in both FAERS and JADER databases was infectious pneumonia adverse events (IPAE), accounting for 9.64 % and 16.19 %, respectively. COVID-19 was included in the number of adverse event reports, accounting for 1.90 % and 0.53 %, respectively, as detailed in Table 4.

**Table 4:** Basic information on infection-related adverse effects.

Characteristics		IRAE					
		FAERS			JADER		
		Subgroups	Cases/N	Proportion/%	Subgroups	Cases/N	Proportion/%
Cases		All	13,946	100.00	All	951	100.00
	Location of infection	Respiratory infection	5499	39.43	Respiratory infection	312	32.80
		Gastrointestinal infection	1846	13.24	Gastrointestinal infection	162	17.04
		Other	6601	47.33	Other	477	50.16
	Pathogenic microorganisms	Bacteria	4778	34.26	Bacteria	426	44.79
		Virus	1468	10.53	Virus	91	9.57
		Fungus	313	2.24	Fungus	21	2.21
		Other	7387	52.97	Other	413	43.43
Infection (Top 10)		Infectious pneumonia	1345	9.64	Infectious pneumonia	154	16.19
		Nasopharyngitis	864	6.19	Sepsis	88	9.25
		Urinary tract infection	686	4.92	Pseudomembranous colitis	47	4.94
		Sepsis	503	3.61	Infective shock	39	4.10
		Bronchitis	490	3.51	Clostridium difficile infection	31	3.26
		Influenza	463	3.32	Difficult fusiform spores	28	2.94
		Nasal sinusitis	415	2.98	Infectious aspiration pneumonia	27	2.84
		Clostridium difficile infection	370	2.65	Phlegmon	25	2.63
		Renal infection	369	2.65	Esophageal moniliasis	19	2.00
		COVID-19	276	1.9	COVID-19	5	0.53

COVID-19 = Corona Virus Disease 2019, FAERS** **=** **the U.S. Food and Drug Administration adverse event reporting system, IRAE = infection-related adverse effects, JADER = the Japan adverse drug event report database, N = number.

**Figure 3.**
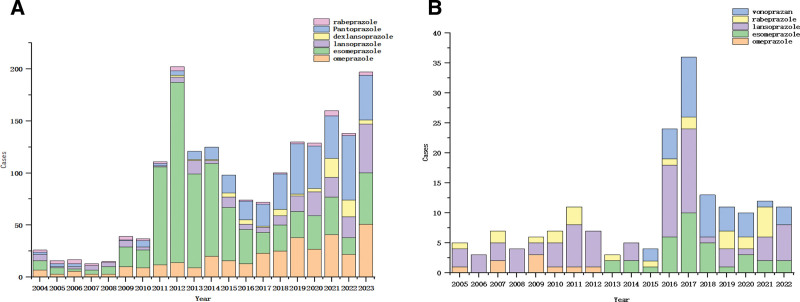
(A) Time distribution of IPAE in the FAERS Database, (B) time Distribution of IPAE in the JADER Database. FAERS = FDA adverse event reporting system, JADER = Japan adverse drug event report database, IPAE = infectious pneumonia adverse events.

IPAE (infectious pneumonia, Infectious aspiration pneumonia and COVID-19 included) is the ADE with the highest occurrence of IRAE in the 2 databases, and its occurrence time is analyzed. In the FAERS database, the incidence of IPAE began to increase significantly from 2011, and began to decline after reaching the highest value in 2012. Until the outbreak of COVID-19 in 2019, its incidence began to increase continuously. Among them, esomeprazole was the PPIs with the highest incidence of IPAE during 2011 to 2014. After 2017, pantoprazole and omeprazole were significantly higher than other PPIs, as shown in Figure 3A. In the JADER database, the incidence of IPAE was not high after the use of PPIs before 2015. After the listing of vonoprazan in Japan in 2015, the incidence of IPAE increased sharply during 2016 to 2017, and stabilized after 2018. Among them, lansoprazole has the largest proportion of IPAE, as shown in Figure 3B.

The IRAE signals after the second screening of PPIs were statistically analyzed, and the MedDRA (SOC) was used to classify the PT with signals, and finally the effective signal PT and the involved system (SOC) were obtained. All PPIs signals were detected and obvious IRAE signals were detected. A total of 144 infected ADRs signals were detected in the FAERS database, involving 2 758 reports, and 31 ADRs signals were detected in the JADER database, involving 194 reports. The signal intensity of ADRs (ROR = 6.39, 95%CI: 5.54–7.37) was higher than that of FAERS (ROR = 2.62, 95%CI: 2.53–2.72). Then we performed signal detection on individual PPIs, and significant IRAE signals were detected in all PPIs. In the FAERS database, the signal detected by lansoprazole was the strongest (ROR = 5.51, 95%CI: 5.06–5.99), PRR was 5.51 (χ^2^ = 1969.08), followed by pantoprazole (ROR = 4.41, 95%CI: 4.07–4.77), PRR was 4.41 (χ^2^ = 1603.67), and the signal detected by esomeprazole was the weakest (ROR = 1.66, 95%CI: 1.56–1.77). In the JADER database, rabeprazole had the strongest signal (ROR = 15.97, 95%CI: 7.90–32.27), PRR was 7.89 (χ^2^ = 108.82), followed by vonoprazan (ROR = 15.23, 95%CI: 12.19–19.04), PRR was 15.19 (χ^2^ = 1028.01), omeprazole had the weakest signal (ROR = 4.87, 95%CI: 2.88–8.25), PRR was 4.86 (χ^2^ = 42.75), as shown in Table 5.

**Table 5:** Signal detection of IRAE in databases.

Subgroups		Cases	IRAE signal/n	IRAE reports/n	Proportion/%	ROR	95% CI	PRR	*χ* ^2^
Infection	FAERS	All	2758	13,946	19.78	2.62	2.53–2.72	2.62	2751.38
		Omeprazole	548	2806	19.53	2.95	2.71–3.21	2.95	702.27
		Esomeprazole	986	6004	16.42	1.66	1.56–1.77	1.66	257.51
		Lansoprazole	537	1652	32.51	5.51	5.06–5.99	5.51	1969.08
		Dexlansoprazole	27	297	9.10	3.79	2.60–5.52	3.78	55.28
		Pantoprazole	614	2922	21.01	4.41	4.07–4.77	4.41	1603.67
		Rabeprazole	46	265	17.36	3.79	2.84–5.06	3.79	94.39
	JADER	All	194	951	20.40	6.39	5.54–7.37	6.38	859.65
		Omeprazole	14	79	17.72	4.87	2.88–8.25	4.86	42.75
		Esomeprazole	20	137	14.60	5.62	3.62–8.74	5.61	75.08
		Lansoprazole	70	414	16.91	6.44	5.08–8.18	6.44	310.60
		Rabeprazole	8	117	6.84	15.97	7.90–32.27	7.89	108.82
		Vonoprazan	82	204	40.20	15.23	12.19–19.04	15.19	1028.01

FAERS = the U.S. Food and Drug Administration adverse event reporting system, IRAE = infection-related adverse effects, JADER = the Japan adverse drug event report database, PRR = the proportional reporting ratio, ROR = the reporting odds ratio, χ^2^ = the chi-square.

## 4. Discussion

With the widespread use of PPIs, its related ADRs has attracted the attention of health professionals, the public and the media. Among them, the increased risk of fracture, acute and chronic kidney disease, gastrointestinal infection and magnesium deficiency has been reported.^[22]^ while there are few reports of COVID-19 and respiratory infections. Respiratory infections are considered to be a possible risk associated with long-term use of PPIs. Several studies have suggested that the mechanism by which the use of PPIs leads to pneumonia may be multifactorial, firstly the increase in bacterial fixation in the stomach due to the elevation of gastric pH caused by PPIs treatment may ultimately lead to an increased risk of pneumonia^[14]^; Secondly PPIs can alter the normal gastrointestinal and oropharyngeal flora, and trace inhalation of this microbiota may lead to pneumonia^[23,24]^;and finally PPIs have been been determined to be present in the respiratory tract and their use may alter the microbiota in the respiratory tract, which may induce pneumonia.^[25]^Currently, there are insufficient clinical trial data to determine if there is an association between PPIs and pneumonia.

This study investigated the post-marketing ADE reports of 6 PPIs (omeprazole, esomeprazole, lansoprazole, dexlansoprazole, pantoprazole and rabeprazole) and potassium ion blockers (vonoprazan), and focused on the occurrence of infectious pneumonia after the use of PPIs. This is the first pharmacovigilance study on the relationship between PPIs and IRAE based on FAERS and JADER databases. The results showed that in the FAERS database, more than half of the cases of PPIs adverse events were reported by non-health professionals, and less than half of the cases were reported by physicians and pharmacists. even among consumers. In JADER, physician-reported cases accounted for 65.51 %. The report age <18 years old included in this study was <2 %, which may be related to the fact that PPIs instructions are not recommended for children.

In both databases, it is shown that the number of people suffering from IRAE after using PPIs is mainly middle-aged and elderly people over 45 years old, which may be the reason for the decline of resistance in middle-aged and elderly people. The systems involved in the adverse reactions mentioned in the instructions, such as digestive system, nervous system, urinary system, musculoskeletal system, etc, were detected in this study, which confirmed the reliability of this study. However, the instructions did not mention the adverse reactions of infectious pneumonia. In this study, new signals of infectious pneumonia were detected, which may be newly discovered ADRs or new common ADRs. Further research is needed to provide reference for the improvement of the instructions.

Both databases in this study suggest that the most SOC distribution of PPIs adverse events is gastrointestinal diseases, which is consistent with previous research results.^[26]^

IRAE was analyzed in detail and it was found that it was mainly respiratory tract infection caused by bacteria. In both databases, infectious pneumonia ranked first and novel coronavirus pneumonia ranked tenth. In the analysis of infectious pneumonia, it was found in the FAERS database that IPAE peaked in 2012 and declined sharply after 2013. Considering that it was related to the sharp increase in the number of adverse reactions in the previous year, it could attract the attention of countries and the masses, and introduce relevant documents to control the use of PPIs, or cause medical staff and patients to change their views on PPIs, and be cautious about the use of PPIs. Therefore, its use rate dropped sharply, and the number of reported adverse reactions also decreased. However, after 2019, IPAE began to rise again. Considering the outbreak of COVID-19, patients diagnosed with COVID-19 will have various digestive symptoms, with a prevalence of 15 %, such as nausea, vomiting, diarrhea and loss of appetite. In addition, patients with gastrointestinal manifestations have poor disease outcomes.^[27]^ The annual distribution of PPIs-related ADE is often related to the use of drugs in the current year and the long-term ADE of PPIs. Therefore, after the outbreak of COVID-19, the use of PPIs has risen sharply, and ADE has also increased. In the JADER database, it was found that IPAE rose sharply in 2016 and peaked in 2017. It was found in the FAERS database that the signal intensity of lansoprazole-related IPAE was the strongest, that is, the correlation between drugs and adverse reactions was high. It was considered that the possibility of lansoprazole-related IPAE was higher than that of other PPIs. In the JADER database, it was found that the intensity of IPAE signals related to rabeprazole and vonoprazan was similar, indicating that the correlation between these 2 drugs and adverse reactions was similar. Unlike FAERS, lansoprazole was not listed in Japan, so the 2 databases could not be compared. The findings of this study and the available evidence should arouse vigilance against PPIs. PPIs should be prescribed or sold according to strict indications, low doses, and short courses of treatment.^[28]^ Patients with no or unknown indications should discontinue PPIs treatment, and patients with indications should avoid long-term use.

## 5. Limitation

Our study revealed a significant association between PPIs and infectious pneumonia based on the FAERS and JADER databases. However, there are some limitations as follows: First, like other spontaneous reporting systems, both databases are voluntary and open to health professionals and the public, so under-reporting, overreporting or missing information is inevitable. Secondly, the research results are subject to the defects of the denominator, overreporting, or under-reporting, which may cause certain deviations in the results; in addition, the number of cases using onset time analysis is limited. Since the treatment period of PPIs is generally long-term treatment, it is difficult to investigate the date of PPIs use, so it is difficult to evaluate the onset time. Finally, there are many factors of infectious pneumonia, such as influenza and viral infection. This study only analyzes infectious pneumonia, infectious aspiration pneumonia and novel coronavirus pneumonia, so there is a certain deviation in the results.

## 6. Conclusion

The present study analyzed the FAERS and JADER databases for adverse event reports from 2004 to 2023, focusing on infectious pneumonias, and used the ROR and MHRA methods of the proportional imbalance method for data mining of adverse events in PPIs, and the consistency of the data with the specification proved the reliability of the present study, which is a reference for the safe use of medication in the clinic. However, the information mined from the database is for informational purposes only and does not necessarily represent actual medication use, and it is expected that pharmacology and basic research in the future will provide high-quality safety evidence to guide and standardize the clinical rational application of PPIs drugs.

## Author contributions

**Writing – original draft**: Shanshan Hu, Zhi Liu.

**Writing – review & editing**: Hongxiu Yu.

## Correction

This article was originally published with an incorrect affiliations for Hongxiu Yu and Zhi Liu. The incorrect affiliations have now been corrected in the online version, and the corrected affiliations appears as “Department of Pharmacy, Panjiang Coal and Electricity Group Hospital, Liupanshui, Guizhou Province, China” for Author “Hongxiu Yu” & “Department of Dermatology, Affiliated Hospital of Guizhou Medical University, Guiyang, Guizhou Province, China” for Author “Zhi Liu.”
